# Supplementary material for: Structural Analysis of the SANT/Myb Domain of FLASH and YARP Proteins and Their Complex with the C-Terminal Fragment of NPAT by NMR Spectroscopy and Computer Simulations
Source: Int J Mol Sci. 2020 Jul 24;21(15):5268. doi: 10.3390/ijms21155268 (PMC7432317; doi:10.3390/ijms21155268)
Supplement: Supplementary file 1 [file ijms-21-05268-s001.zip › SupportingMaterials/flash_yarp_sequence-eps-converted-to.pdf]

```
FLASH
1923 GEIIILWTRNDDREILLECQKRGPSFKTFAYLAAKL-DKNPNQVSERFQQLMKLFEKSKCR 1982
     GE ++LWTR  DR IL  CQ++G   +TF ++  +L +K P +VS RF++LM+LF  +
2149 GEKVVLWTREADRVILTMCQEQGAQPQTFNIISQQLGNKTPAEVSHRFRELMQLFHTACEA 2209
YARP
```
