# Supplementary figures and images for: Structural Analysis of the SANT/Myb Domain of FLASH and YARP Proteins and Their Complex with the C-Terminal Fragment of NPAT by NMR Spectroscopy and Computer Simulations
Source: Int J Mol Sci. 2020 Jul 24;21(15):5268. doi: 10.3390/ijms21155268 (PMC7432317; doi:10.3390/ijms21155268)

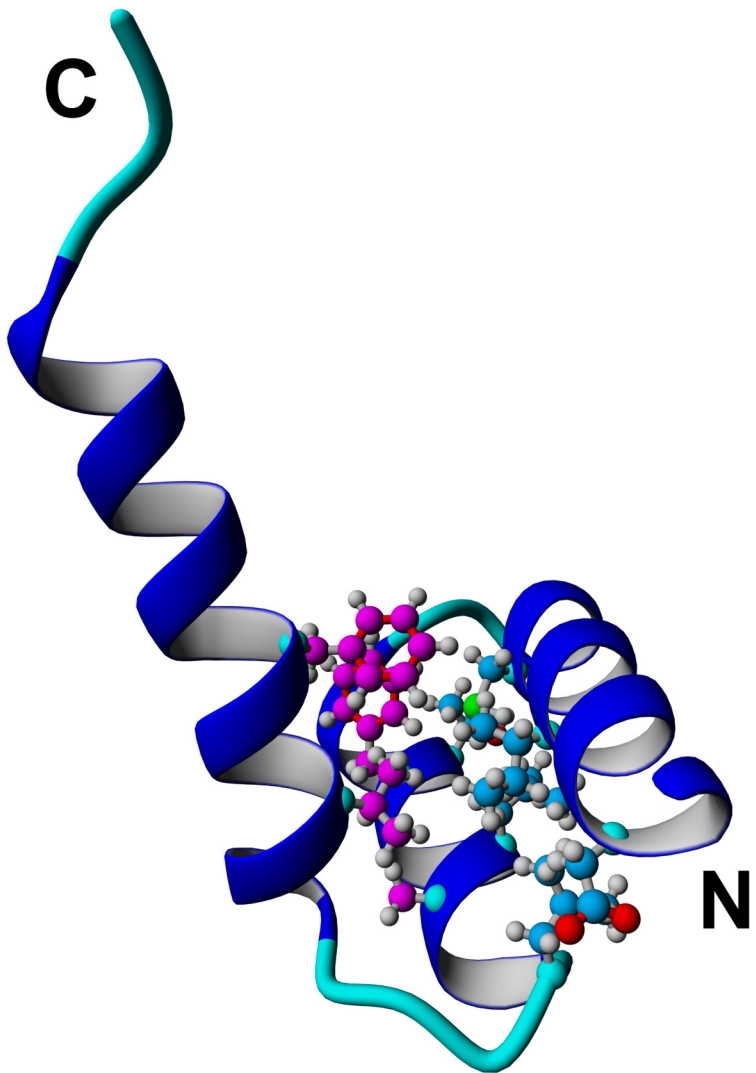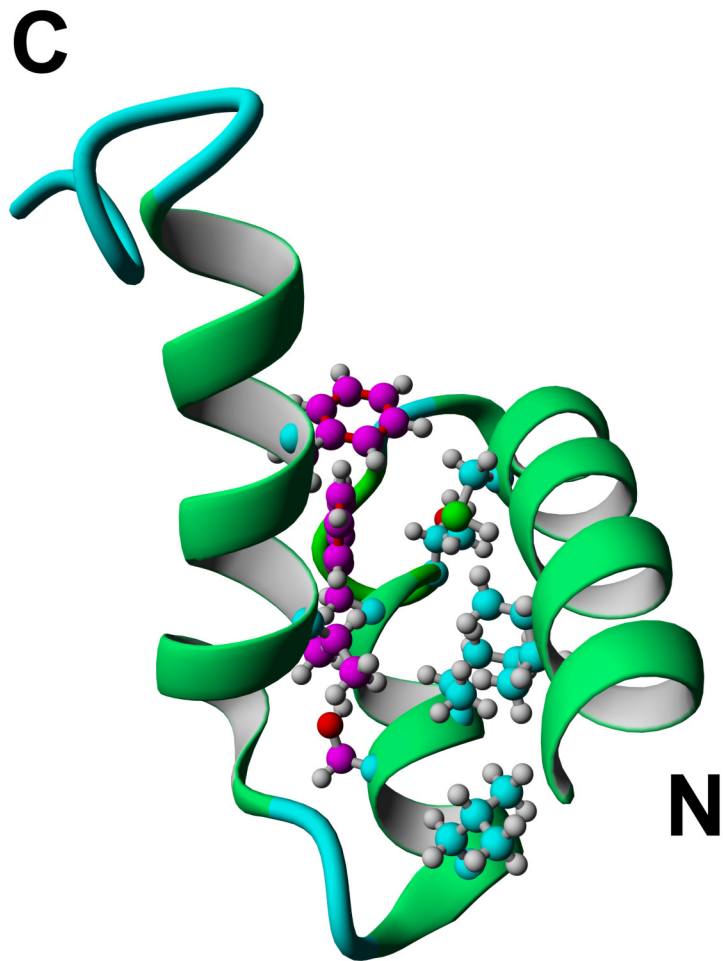

Supplement: Supplementary file 1 [file ijms-21-05268-s001.zip › SupportingMaterials/flash_yarp_contacts_interhelical-eps-converted-to.pdf]

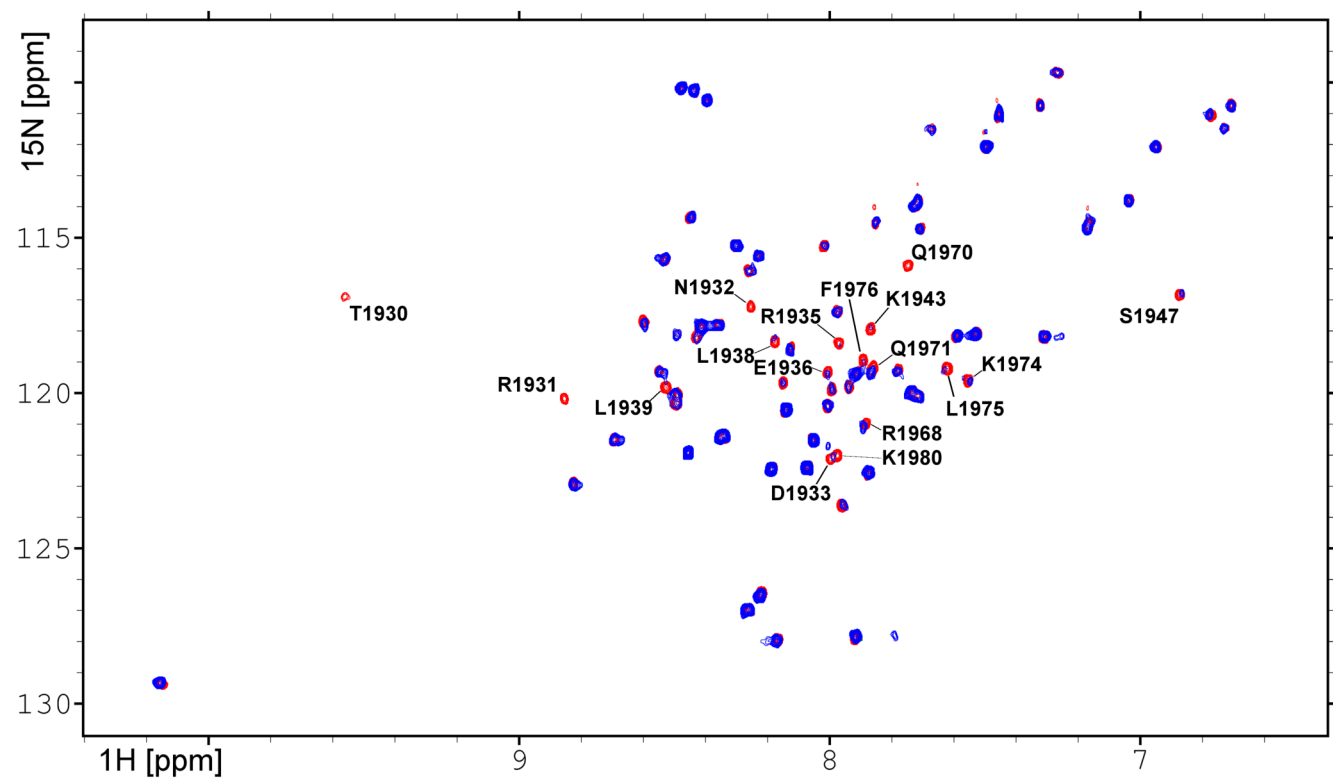

Supplement: Supplementary file 1 [file ijms-21-05268-s001.zip › SupportingMaterials/flash_npat_hsqc_15N-eps-converted-to.pdf]

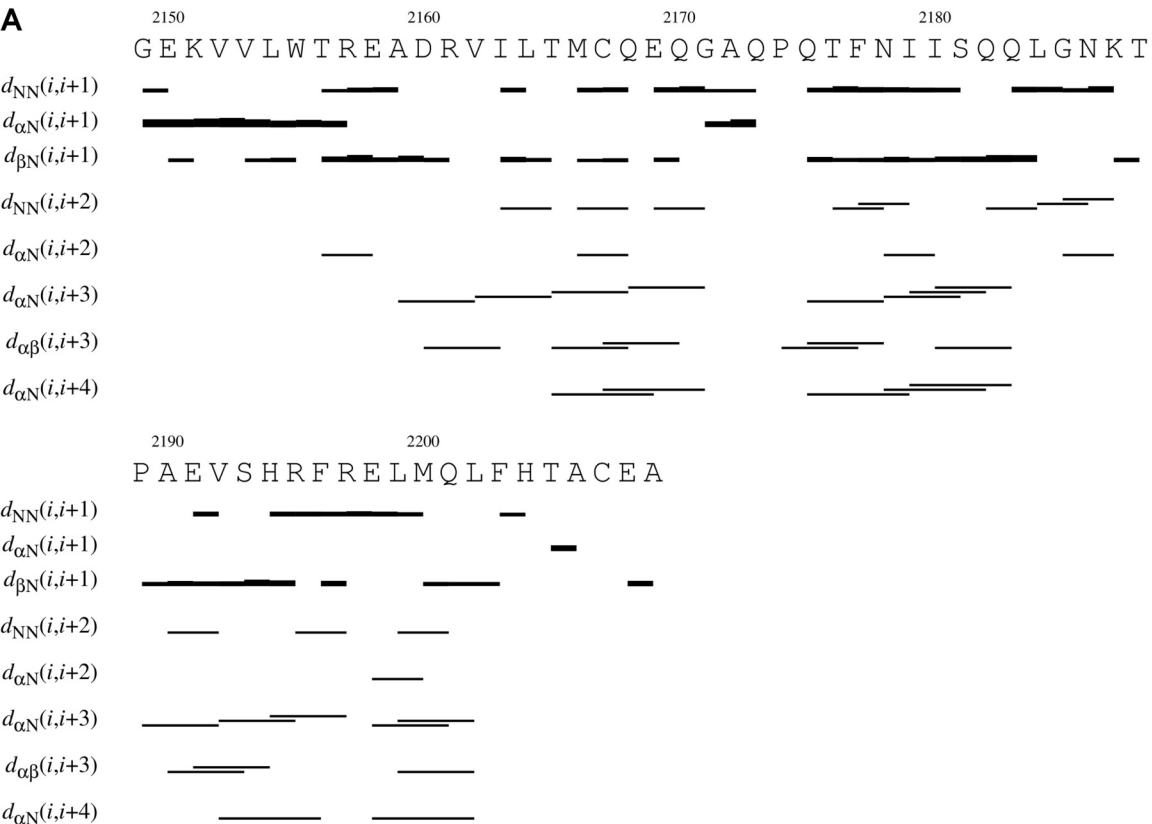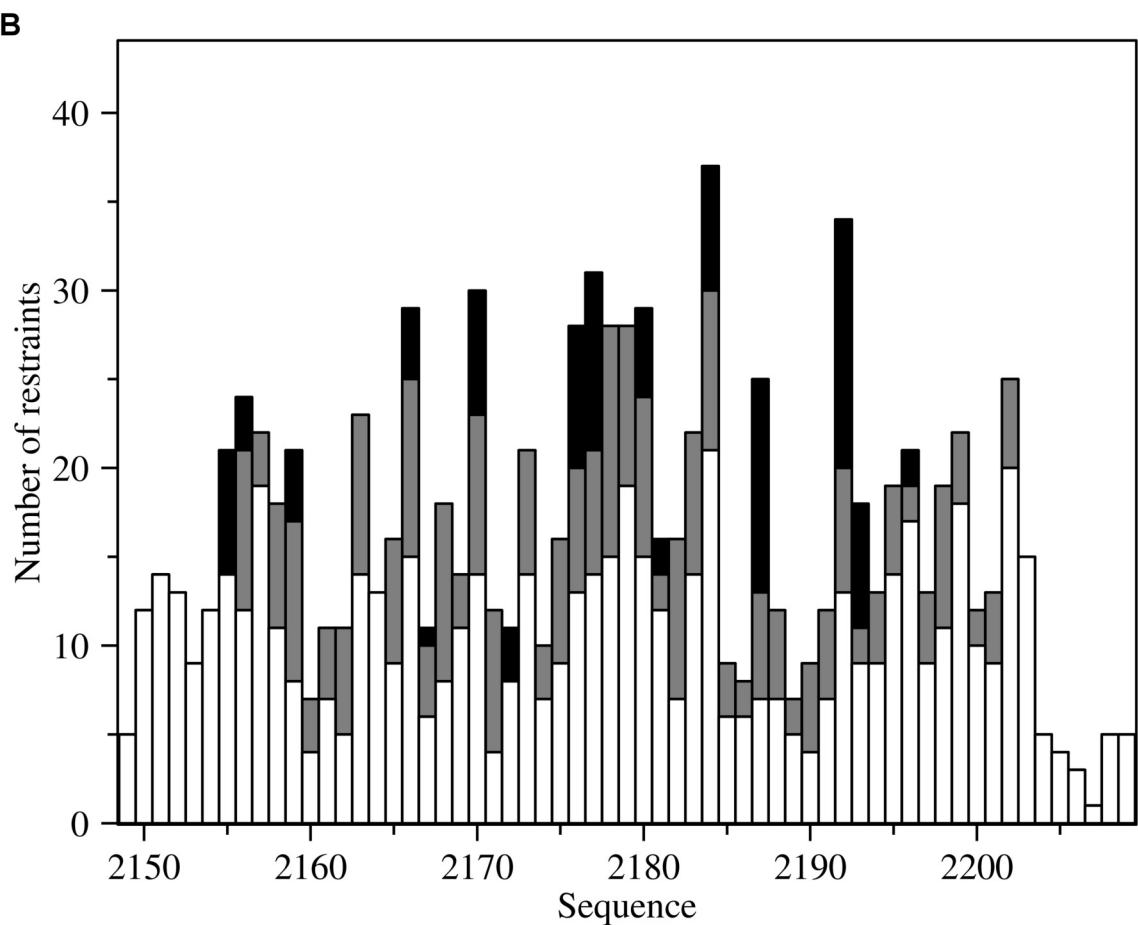

Supplement: Supplementary file 1 [file ijms-21-05268-s001.zip › SupportingMaterials/yarp_cyana-eps-converted-to.pdf]

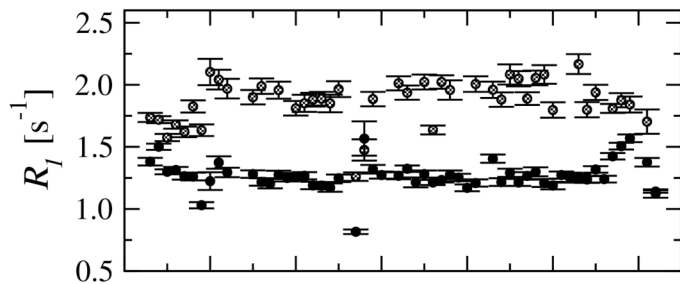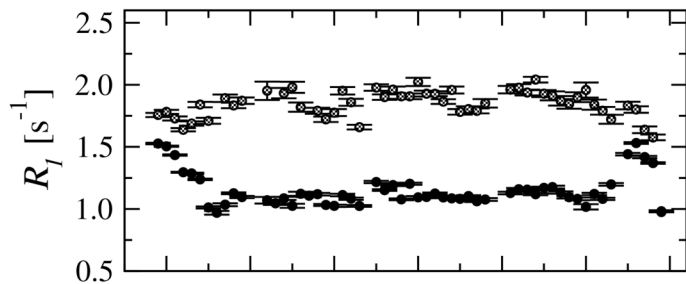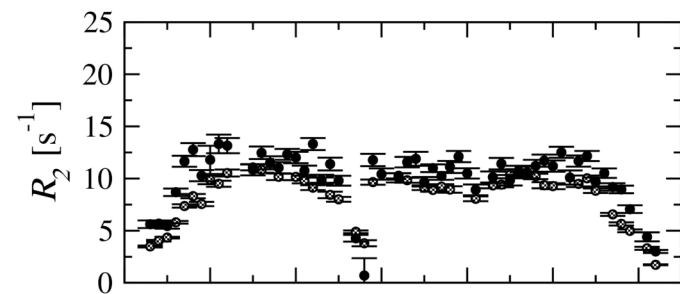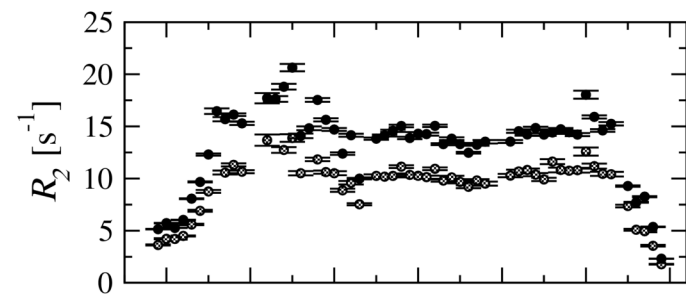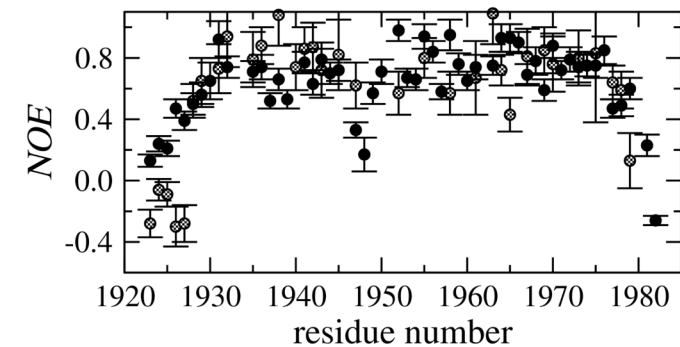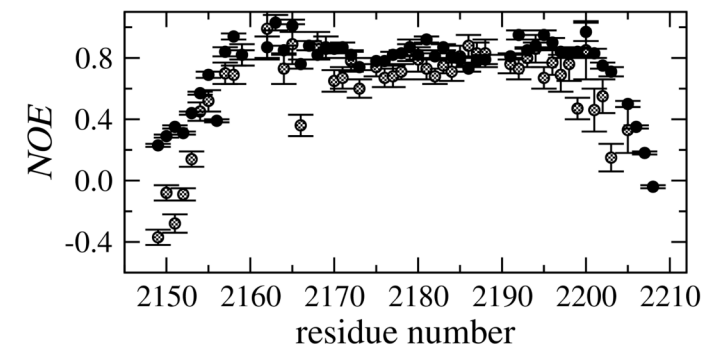

Supplement: Supplementary file 1 [file ijms-21-05268-s001.zip › SupportingMaterials/flash_yarp_relax_data-eps-converted-to.pdf]

**A**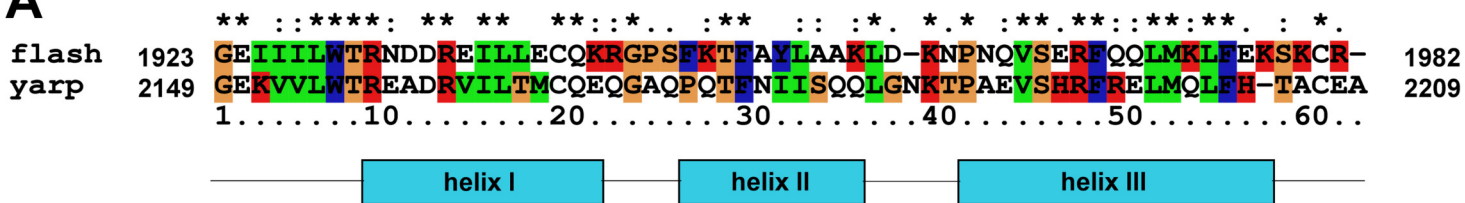**B**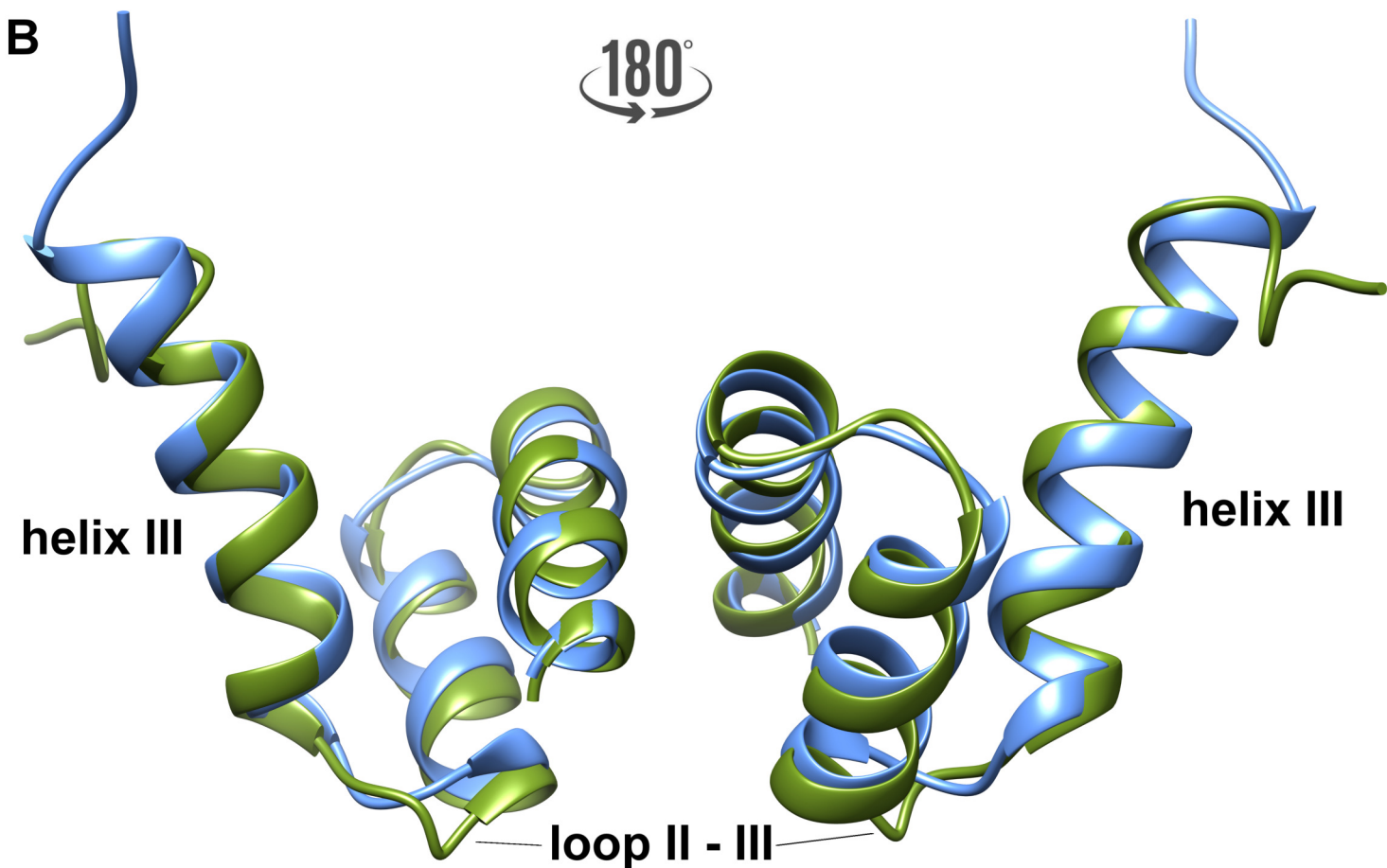

Supplement: Supplementary file 1 [file ijms-21-05268-s001.zip › SupportingMaterials/flash_yarp_alignment_match-eps-converted-to.pdf]

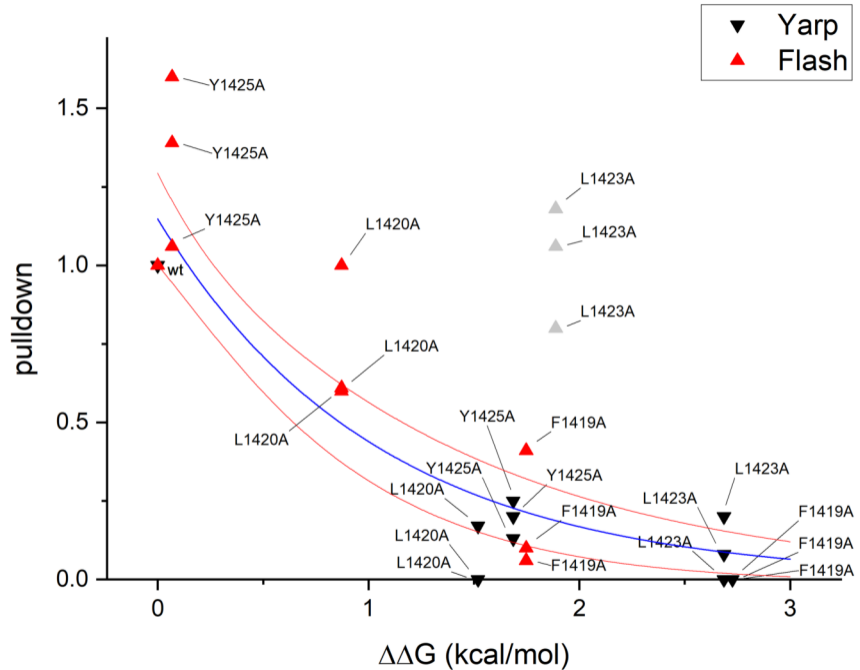

Supplement: Supplementary file 1 [file ijms-21-05268-s001.zip › SupportingMaterials/flash_yarp_pulldown_analysis-eps-converted-to.pdf]

$180^\circ$

helix III

helix III

loop II - III

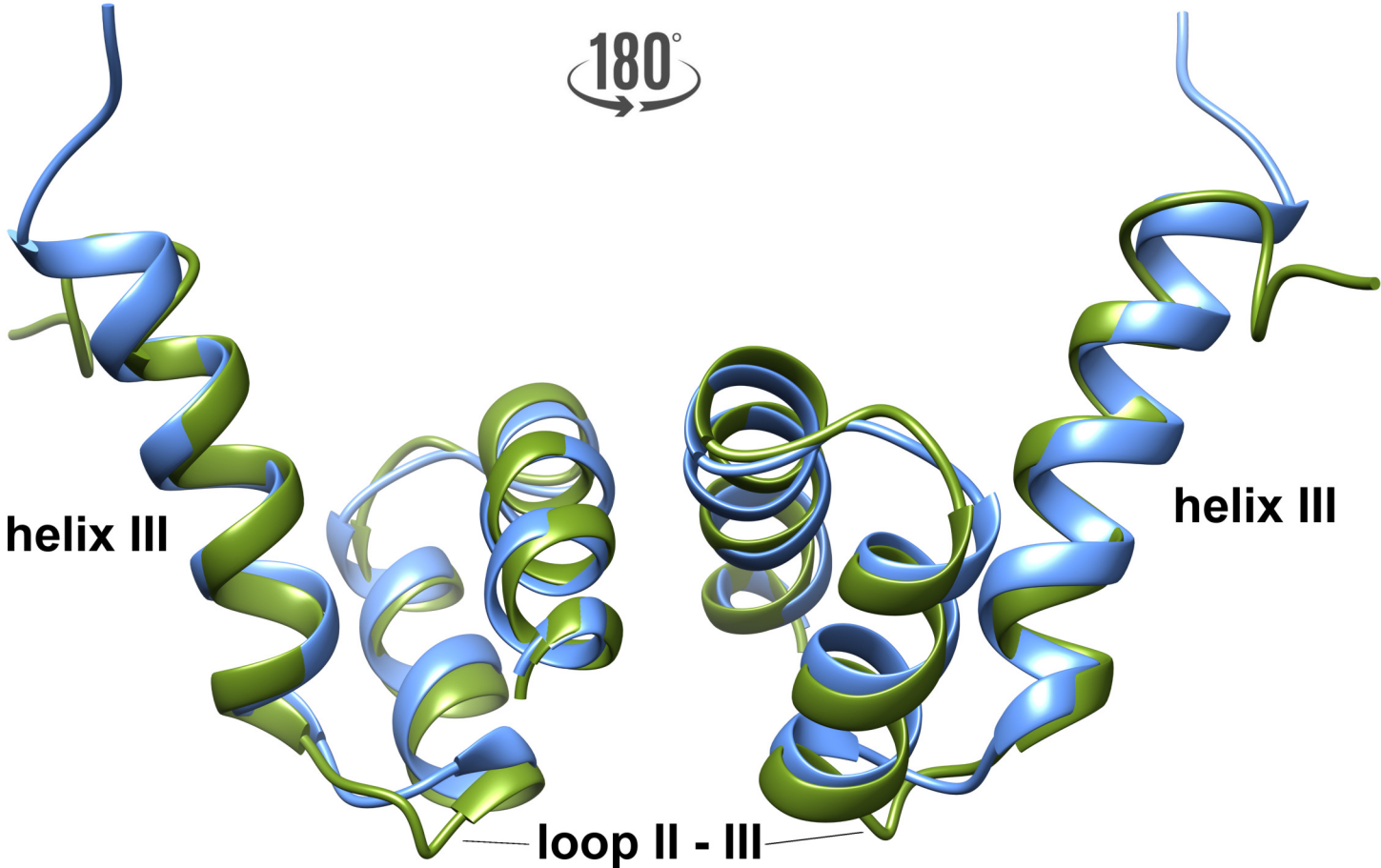

Supplement: Supplementary file 1 [file ijms-21-05268-s001.zip › SupportingMaterials/flash_yarp_match-eps-converted-to.pdf]

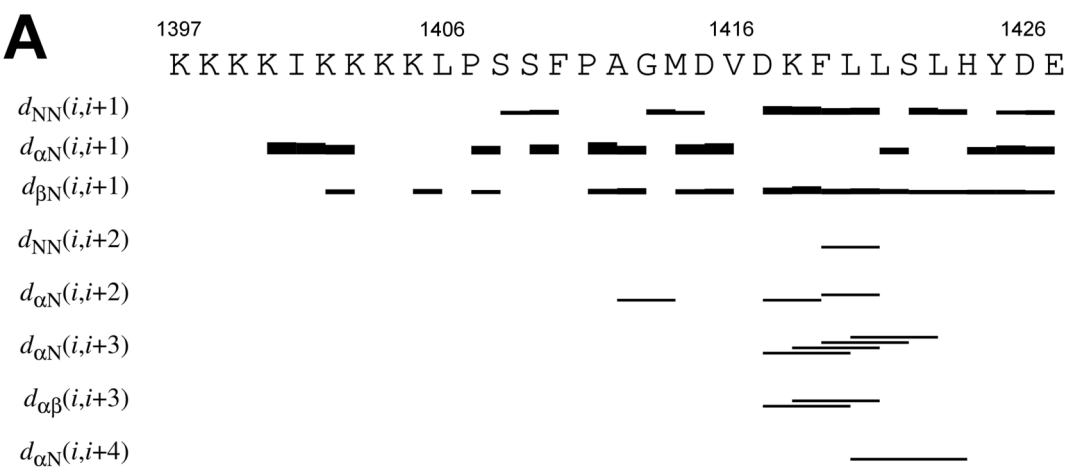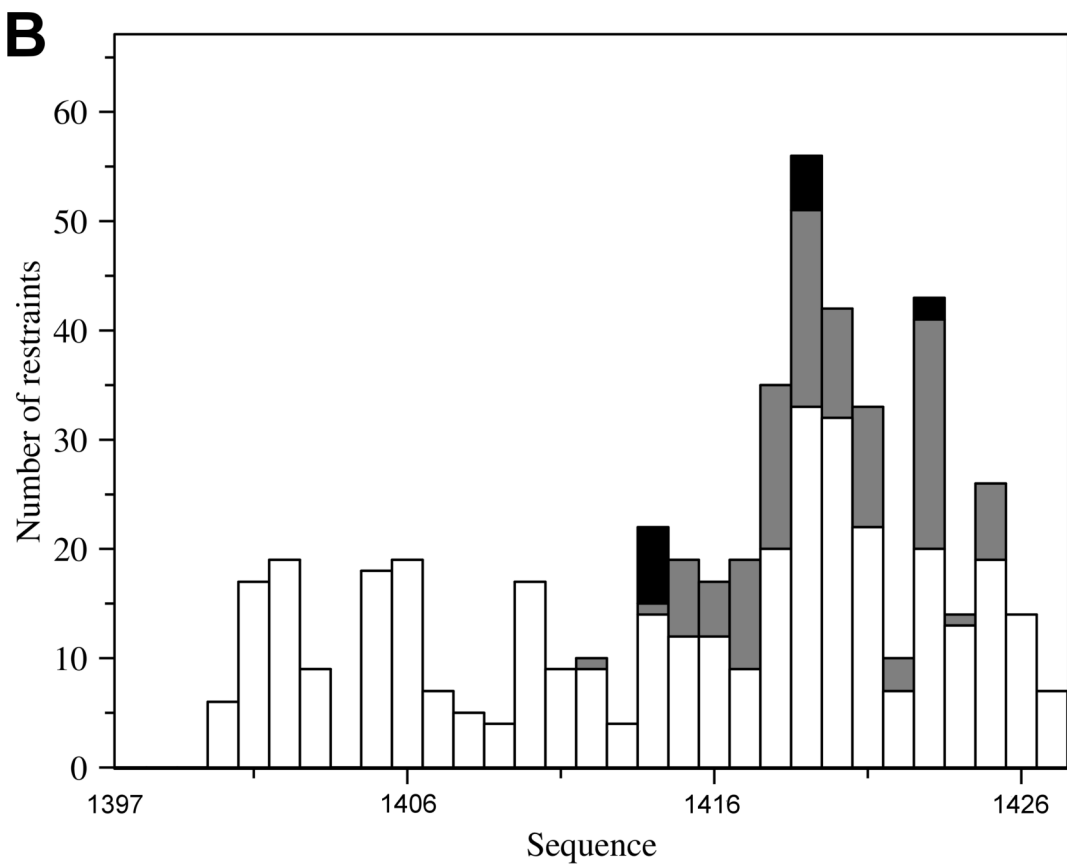

Supplement: Supplementary file 1 [file ijms-21-05268-s001.zip › SupportingMaterials/npat_cyana-eps-converted-to.pdf]

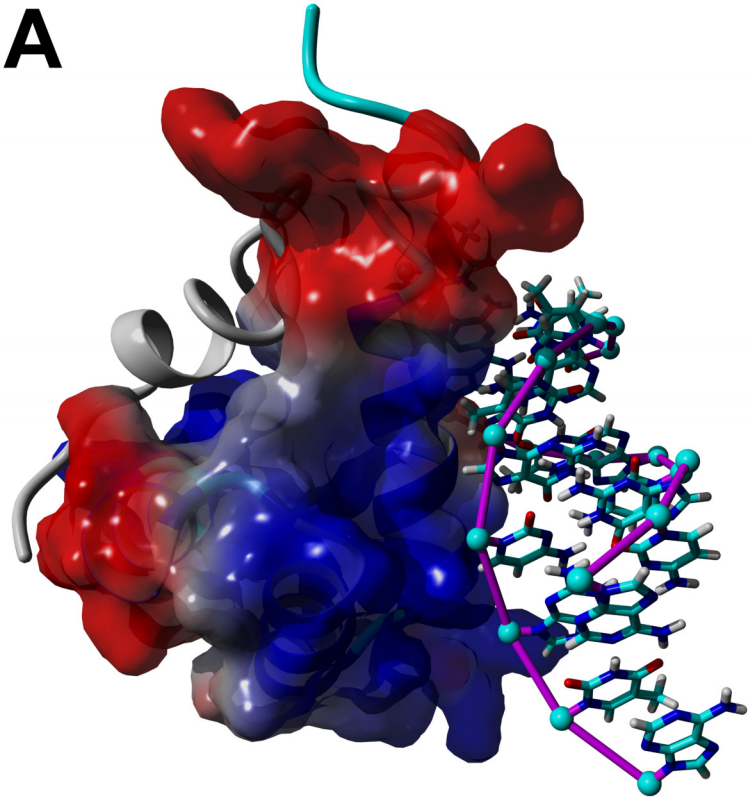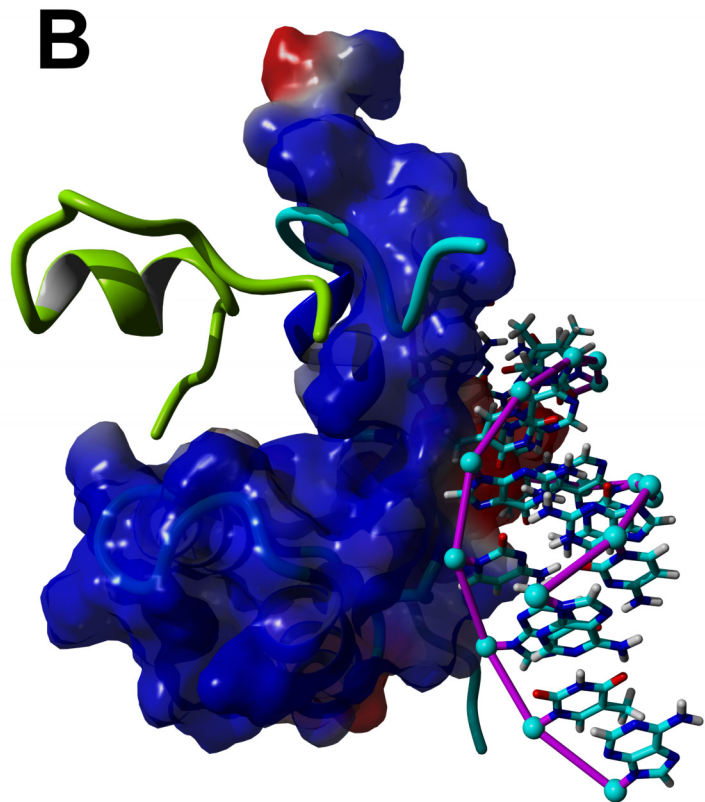

Supplement: Supplementary file 1 [file ijms-21-05268-s001.zip › SupportingMaterials/flash_yarp_npat_dna-eps-converted-to.pdf]

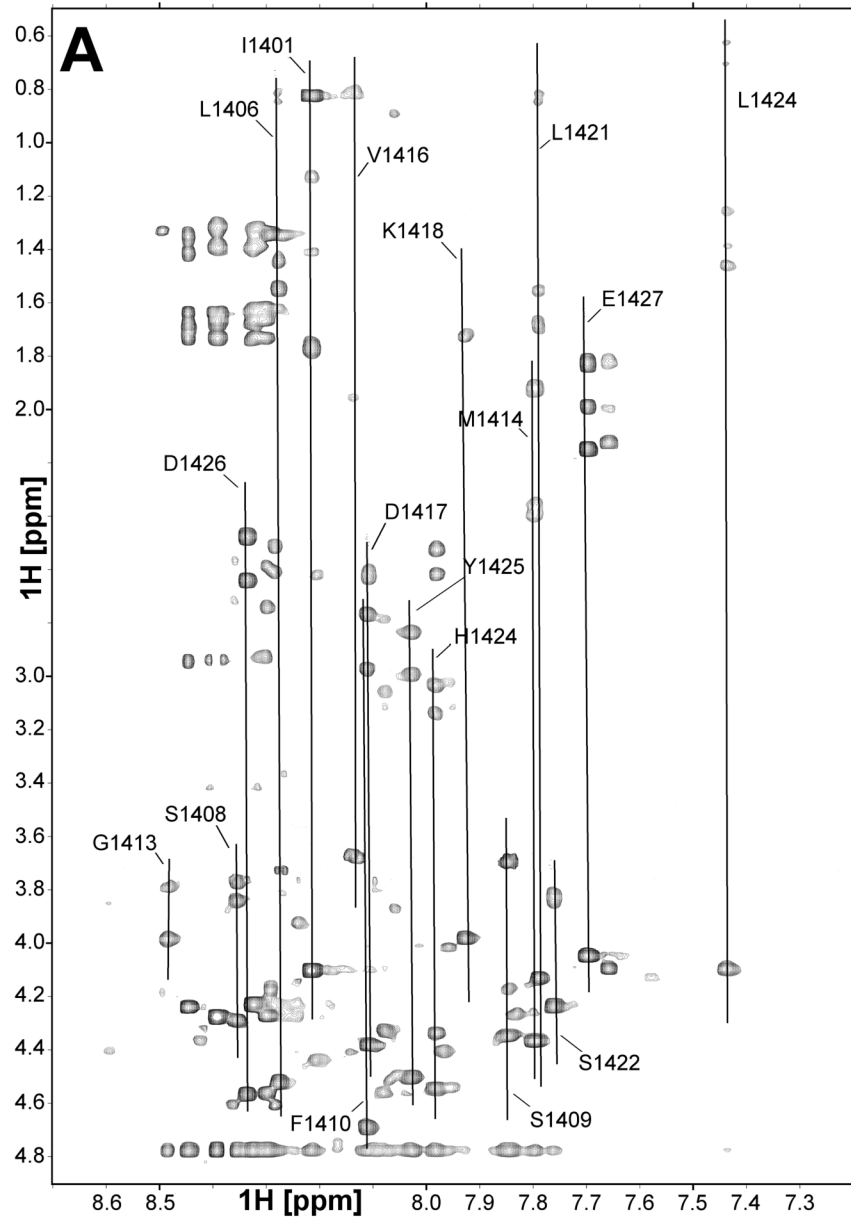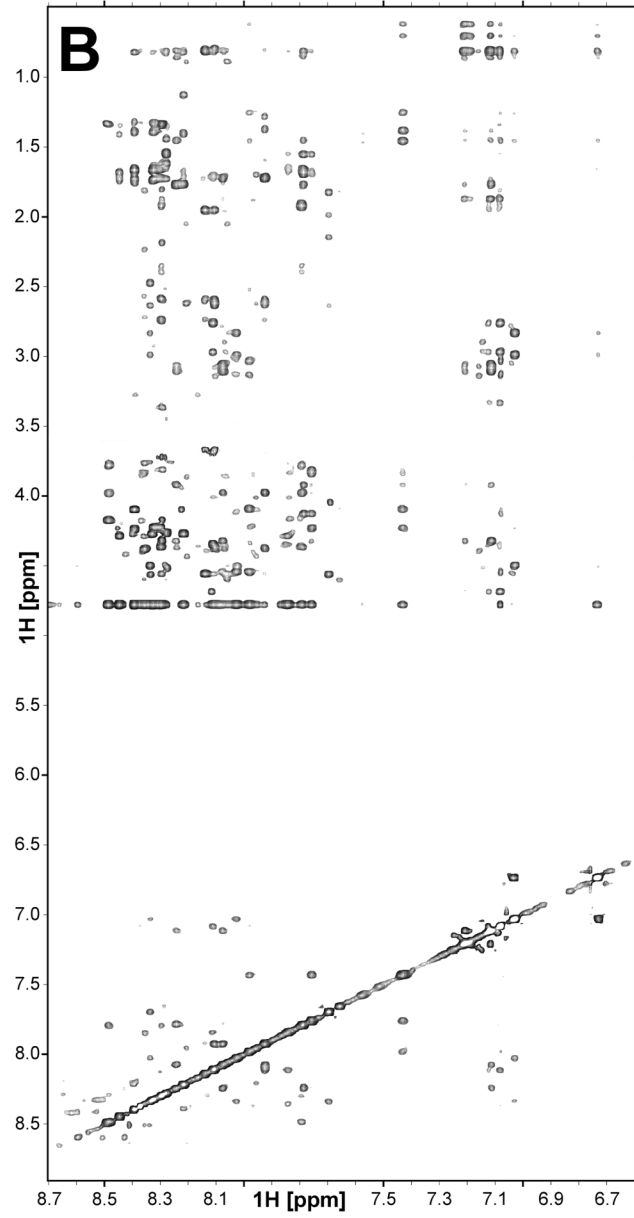

Supplement: Supplementary file 1 [file ijms-21-05268-s001.zip › SupportingMaterials/npat_tocsy_noesy-eps-converted-to.pdf]

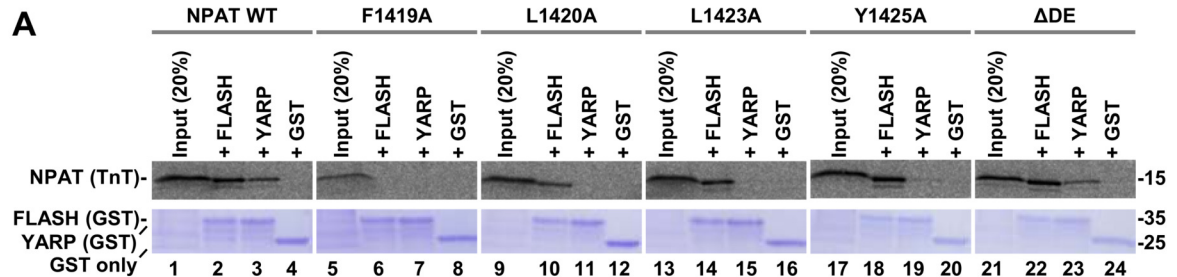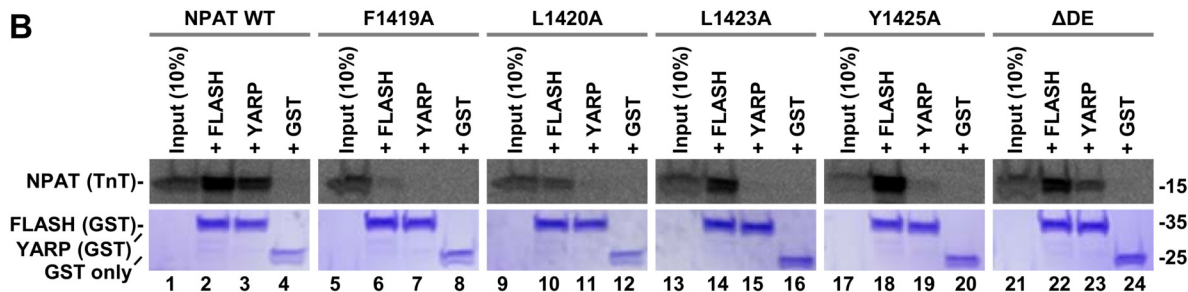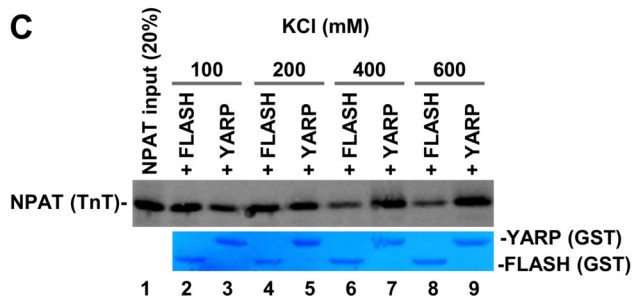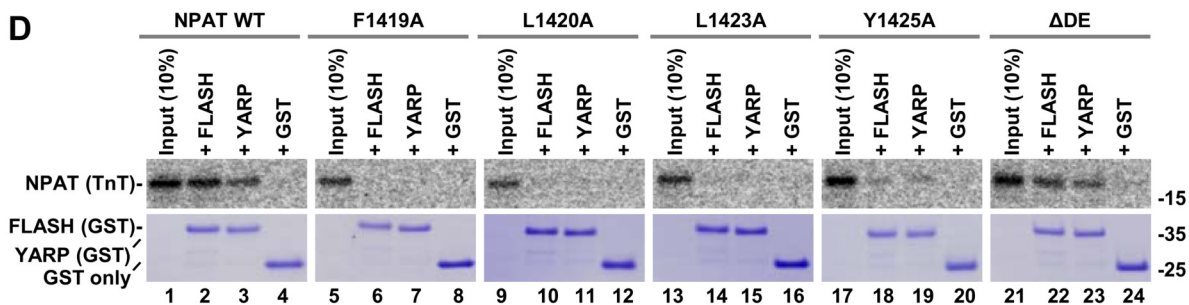

Supplement: Supplementary file 1 [file ijms-21-05268-s001.zip › SupportingMaterials/flash_yarp_npat_mutants-eps-converted-to.pdf]

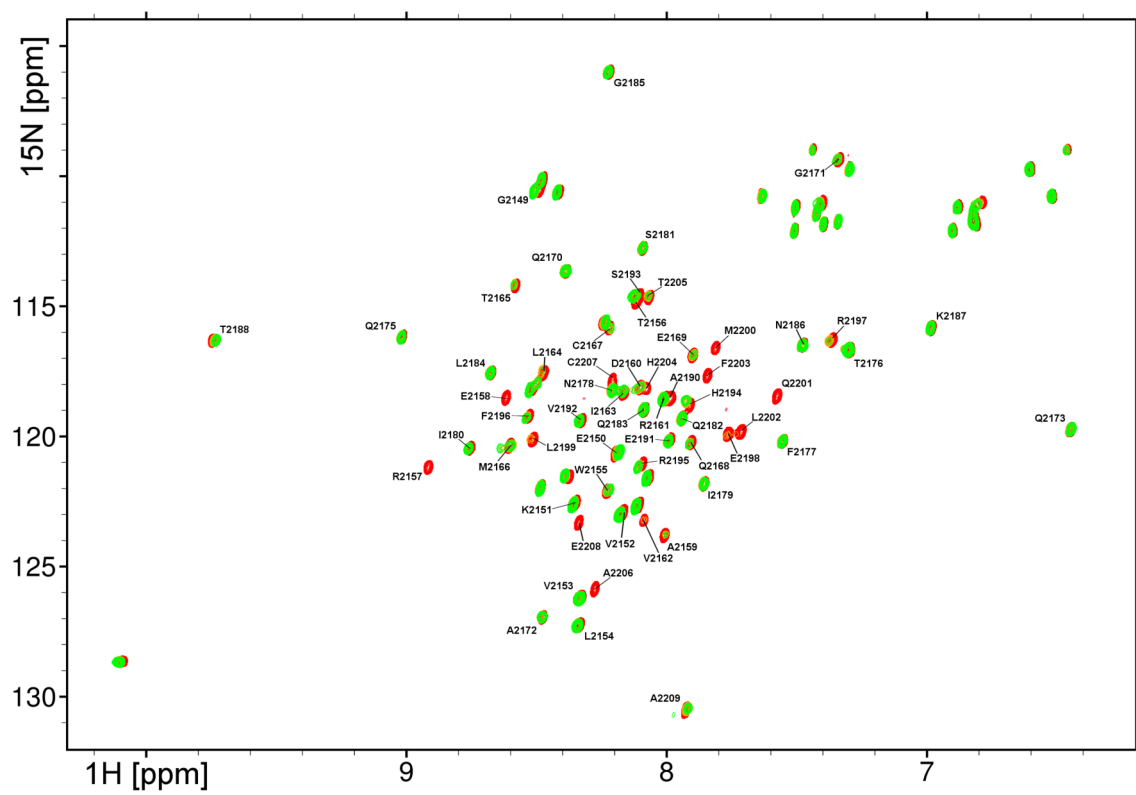

Supplement: Supplementary file 1 [file ijms-21-05268-s001.zip › SupportingMaterials/yarp_npat_hsqc_15N-eps-converted-to.pdf]

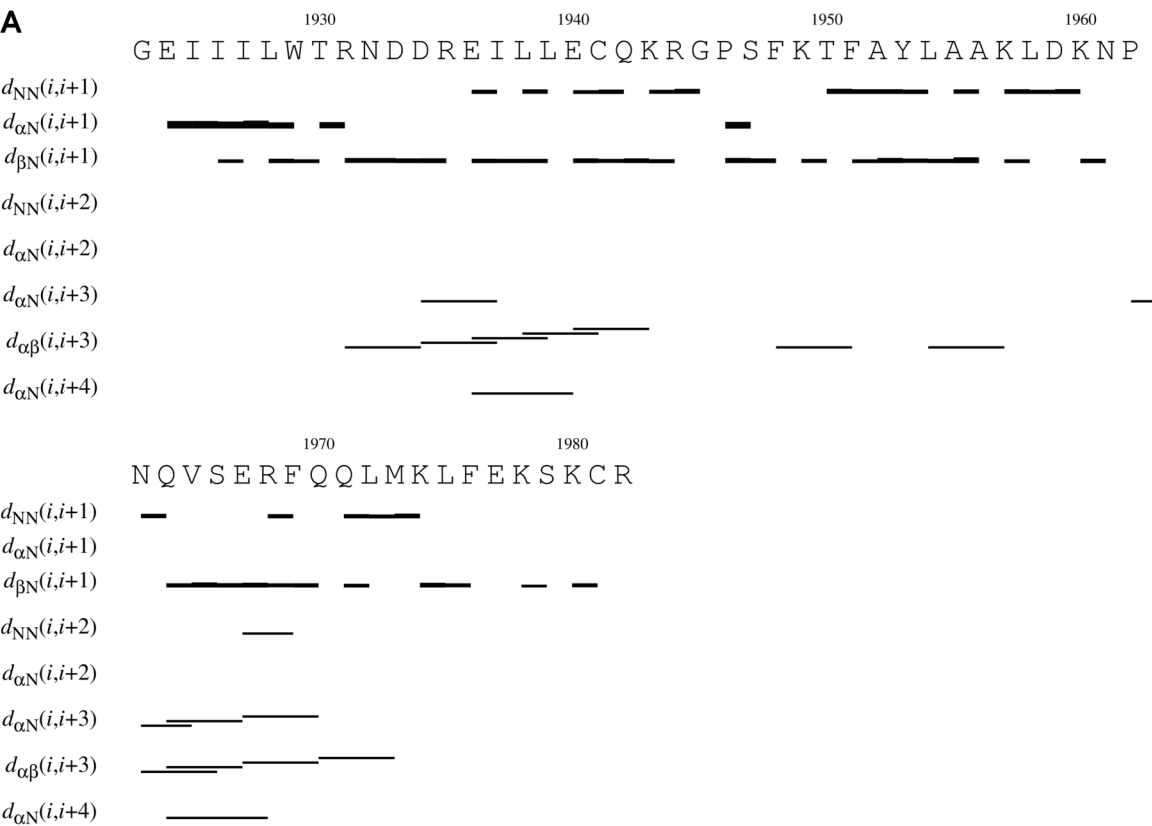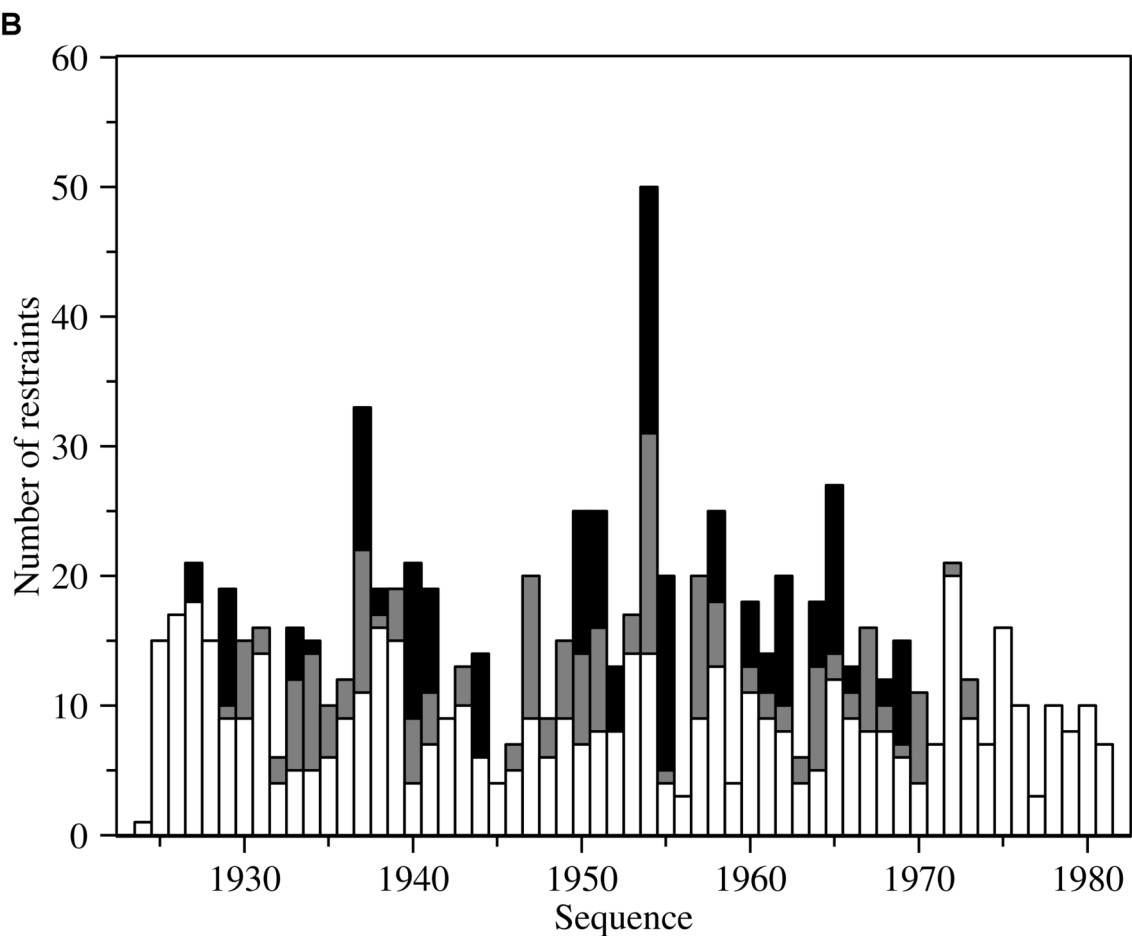

Supplement: Supplementary file 1 [file ijms-21-05268-s001.zip › SupportingMaterials/flash_cyana-eps-converted-to.pdf]

**A**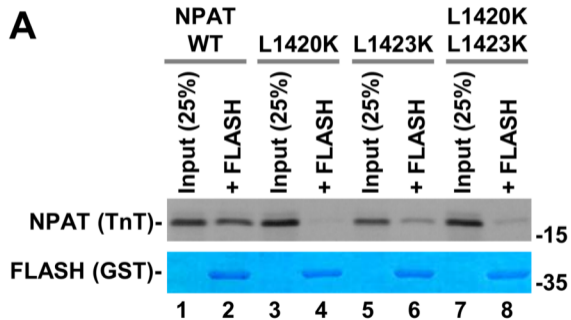**B**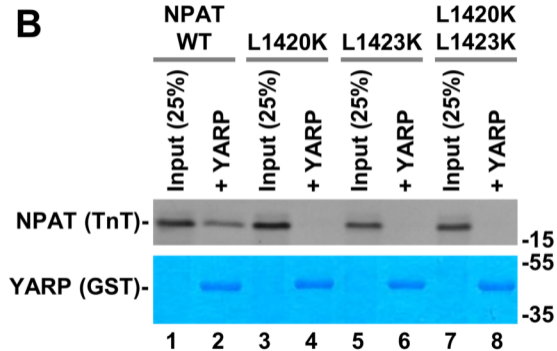

Supplement: Supplementary file 1 [file ijms-21-05268-s001.zip › SupportingMaterials/flash_yarp_npat_gel-eps-converted-to.pdf]
