# Supplementary material for: Structural Analysis of the SANT/Myb Domain of FLASH and YARP Proteins and Their Complex with the C-Terminal Fragment of NPAT by NMR Spectroscopy and Computer Simulations
Source: Int J Mol Sci. 2020 Jul 24;21(15):5268. doi: 10.3390/ijms21155268 (PMC7432317; doi:10.3390/ijms21155268)
Supplement: Supplementary file 1 [file flash_yarp_hsqc_15N-eps-converted-to.pdf › SupportingMaterials/flash_yarp_hsqc_15N-eps-converted-to.pdf]

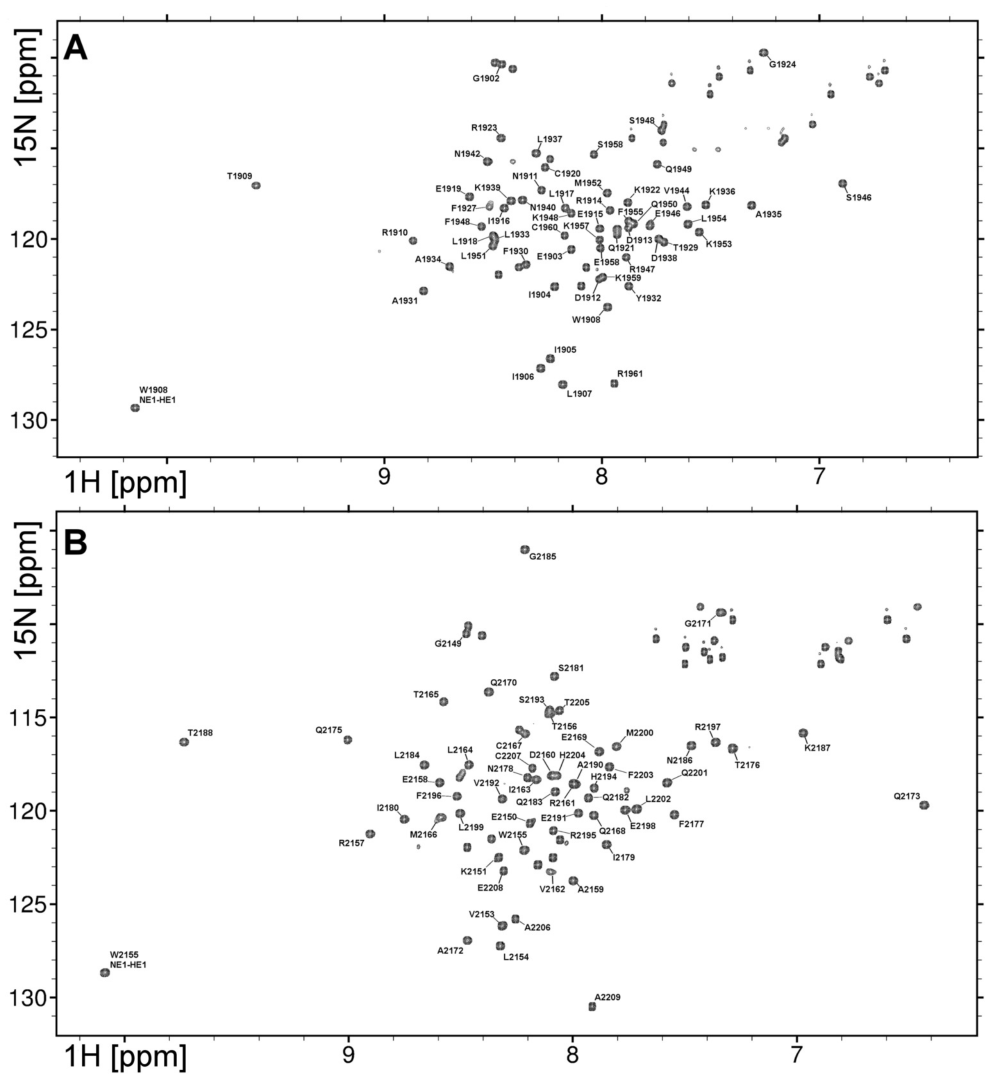
A
15N [ppm]
1H [ppm]
120
125
130
9
8
7
G1902
G1924
S1948
R1923
L1937
S1958
N1942
Q1949
T1909
N1911
C1920
M1952
S1946
E1919
K1939
L1917
K1922
V1944
K1936
R1914
Q1950
F1927
N1940
A1935
F1955
K1948
E1915
E1946
L1954
F1948
I1916
C1960
K1957
R1910
L1918
L1933
D1913
K1953
Q1921
T1929
L1951
F1930
D1938
A1934
E1903
E1958
R1947
K1959
I1904
Y1932
A1931
D1912
W1908
I1905
R1961
I1906
L1907
W1908
NE1-HE1
B
15N [ppm]
1H [ppm]
115
120
125
130
9
8
7
G2185
G2171
G2149
S2181
Q2170
T2165
S2193
T2205
T2188
Q2175
T2156
R2197
E2169
M2200
K2187
C2167
L2164
L2184
D2160
H2204
N2186
T2176
C2207
A2190
N2178
F2203
E2158
V2192
H2194
Q2201
I2163
Q2173
F2196
Q2183
R2161
Q2182
L2202
I2180
E2150
E2191
E2198
F2177
L2199
Q2168
M2166
W2155
R2195
R2157
I2179
K2151
E2208
V2162
A2159
V2153
A2206
A2172
L2154
W2155
NE1-HE1
A2209
